# Use of psychiatric hospitals and social integration of patients with psychiatric disorders: a prospective cohort study in five European countries

**DOI:** 10.1007/s00127-020-01881-1

**Published:** 2020-05-14

**Authors:** Pierre Smith, Pablo Nicaise, Domenico Giacco, Victoria jane Bird, Michael Bauer, Mirella Ruggeri, Marta Welbel, Andrea Pfennig, Antonio Lasalvia, Jacek Moskalewicz, Stefan Priebe, Vincent Lorant

**Affiliations:** 1 Institute of Health and Society IRSS, Université catholique de Louvain, Clos Chapelle-aux-Champs, 30, 1200 Brussels, Belgium; 2 Unit for Social and Community Psychiatry (World Health Organisation Collaborating Centre for Mental Health Services Development), Queen Mary University of London, London, UK; 3 Department of Psychiatry and Psychotherapy, Carl Gustav Carus University Hospital, Technische Universität Dresden, Dresden, Germany; 4 Section of Psychiatry, Department of Public Health and Community Medicine, University of Verona, Verona, Italy; 5 Section of Psychiatry, Department of Neuroscience, Biomedicine and Movement Sciences, University of Verona, Verona, Italy; 6 UOC di Psichiatria, Azienda Ospedaliera Universitaria Intergrata (AOUI) di Verona, Verona, Italy; 7 Institute of Psychiatry and Neurology, Warsaw, Poland

**Keywords:** Mental illness, Social integration, Length of hospital stay, Hospital readmissions, Employment

## Abstract

**Purpose:**

Long lengths of stay (LoS) in psychiatric hospitals or repeated admission may affect the social integration of patients with psychiatric disorders. So far, however, studies have been inconclusive. This study aimed to analyse whether long LoS or repeated admissions in psychiatric wards were associated in different ways with changes in the social integration of patients.

**Methods:**

Within a prospective cohort study, data were collected on 2181 patients with a main ICD-10 diagnosis of psychotic, affective, or anxiety disorder, hospitalised in the UK, Italy, Germany, Poland, and Belgium in 2015. Social integration was measured at baseline and 1 year after admission using the SIX index, which includes four dimensions: employment, housing, family situation, and friendship. Regression models were performed to test the association between LoS, the number of admissions, and the change in social integration over the study period, controlling for patients’ characteristics (trial registration ISRCTN40256812).

**Results:**

A longer LoS was significantly associated with a decrease in social integration (*β* = − 0.23, 95%CI − 0.32 to − 0.14, *p* = 0.03), particularly regarding employment (OR = 2.21, 95%CI 1.18–3.24, *p* = 0.02), housing (OR = 3.45, 95%CI 1.74–5.16, *p* < 0.001), and family situation (OR = 1.94, 95%CI 1.10–2.78, *p* = 0.04). In contrast, repeated admissions were only associated with a decrease in friendship contacts (OR = 1.15, 95CI% 1.08–1.22, *p* = 0.03).

**Conclusions:**

Results suggest that a longer hospital LoS is more strongly associated with a decrease in patients’ social integration than repeated admissions. Special attention should be paid to helping patients to find and retain housing and employment while hospitalised for long periods.

**Electronic supplementary material:**

The online version of this article (10.1007/s00127-020-01881-1) contains supplementary material, which is available to authorized users.

## Introduction

Patients with psychiatric disorders need more than just symptom relief [1]. Most psychiatric patients face social integration difficulties such as unemployment, homelessness, poor social capital, and little social participation in community life [2–4]. There are different definitions of the concepts of social integration and social exclusion in the literature [5, 6]. Social integration is not only limited to the economic participation (production and purchasing capacity) of individuals in society, but also includes dimensions relating to social engagement and social interaction [7, 8]. The social integration of an individual is, therefore, his/her participation in the key activities of the society in which he/she lives (e.g. employment, housing, friendship, etc.). Conversely, a lack of participation in such activities constitutes social exclusion [6, 9]. Over the past few decades, mental health care systems have increasingly considered the social integration of psychiatric patients a major objective and countries have developed policies and interventions aimed at achieving better social integration [2, 5, 10, 11]. However, these initiatives have had mixed results, and the social integration of psychiatric patients remains an issue in high-income countries. For example, in OECD countries, people with psychiatric disorders are still six to seven times more likely to be unemployed than the general population [12]. In regard to housing, the prevalence of homelessness among psychiatric patients is 15%, which is much higher than in the general adult population [13]. The extent to which social exclusion occurs is often greater among patients with more serious mental disorders [14, 15], but it also varies according to the patient’s psychiatric diagnosis. For example, different studies have found an employment rate of between 10 and 20% among people with schizophrenia [16, 17], between 40 and 60% among people with anxiety disorders [18], and up to 60% among people with mood disorders [19].

One of the multiple factors that might hinder the social integration of psychiatric patients is the fact that most of them tend to experience long lengths of stay (LoS) in psychiatric hospitals and repeated admissions, which may disrupt their social and professional lives and affect their social integration, as suggested by Goffman [20], Honigfeld [21], and Gruenberg [22]. In fact, one of the consequences of excessive LoS in psychiatric hospital and psychiatric readmissions is the “social breakdown syndrome”, whereby psychiatric patients experience community and social withdrawal, exclusion from typical social roles, and a decline in their social integration [22, 23]. For example, patients report that there is a stigma attached to psychiatric hospitalisations and fear that they will not be able to return to paid employment after long hospital stays or repeated admissions [24, 25]. Although the average LoS in psychiatric hospitals has decreased in recent decades due to deinstitutionalisation policies, it remains longer than for most physical disorders [26–29]. Some patients with severe and chronic mental illness and complex health and social needs may require longer hospital stays to adapt to psychotropic medication, stabilise symptoms, manage suicidal ideation or aggressive behaviour, and plan hospital discharge and community follow-up [30, 31]. Indeed, some clinical and socio-demographic characteristics of patients, such as having a diagnosis of psychosis, the severity of the illness, or being homeless, have been associated with longer LoS in psychiatric hospitals [26, 32–36]. However, socio-demographic and clinical characteristics of patients predict only 15% of the variance of LoS in psychiatric hospitals [37]. Furthermore, LoS in psychiatric hospitals vary substantially across countries and across hospitals among patients with similar profiles, suggesting that LoS is also determined by features related to policies and practices, i.e. availability of psychiatric hospital beds, legal frameworks for involuntary hospital treatment, availability of alternatives to hospitalisation, funding mechanisms, and the culture of mental health care [32, 38, 39]. In one study, more than 500 long-stay patients (median continuous LoS of more than 20 years), who were discharged from 2 London psychiatric hospitals after their closure in the 1990s [40], were followed-up within the community. The social and clinical outcomes of these patients were assessed during the 5-year period following hospital discharge. The study concluded that there was no change in the severity of patients’ symptoms or social behaviour problems and indicated that there was an improvement in their social capital and community and domestic skills. Taken together, these results indicate that LoS in psychiatric hospitals bears a weak relation to the clinical and social needs of patients at admission, that excessive LoS and repeated admissions may affect patients’ social integration, and that being discharged from hospital may even be beneficial for long-stay patients.

However, the effect of LoS and readmissions on patients’ social integration remains a controversial subject. Some argue that psychiatric hospitalisations should be dedicated to acute care only, should be as short as possible, and should be considered as a last care resort. Long stays in, and repeated admissions to hospital, according to them, disrupt the social and professional lives of patients, have a negative effect on social integration, and lead to institutionalisation [1, 41–43]. According to others, however, an excessive reduction of hospital LoS would lead to precipitous hospital discharge, increasing the likelihood of readmission and inducing a “revolving door” admission phenomenon, which would have adverse effects on patients’ social integration [44–46]. Furthermore, studies analysing the association between lengthy hospital stays or repeated hospitalisations and patients’ social and clinical status have been inconclusive, so it is unclear whether the impact of a long stay in hospital on patients’ social integration would be similar to that of repeated, shorter stays. A systematic review showed that the few studies which have explored such associations have had inconsistent results [25]. Another more recent review of randomised controlled trials [44] comparing the effect of short and long psychiatric hospitalisations showed that patients with serious mental illness who were allocated to short hospital stay interventions had higher post-hospitalisation rates of employment and independent living than patients who were allocated to longer stay interventions. The trials were limited, however, and the quality of the evidence was poor [44]. Other studies showed no significant association between LoS in psychiatric hospitals and patients’ work attendance and employment status after discharge [47, 48].

Both in terms of clinical practice and to support recovery-oriented mental health care, more research is needed on the association that may exist between the use of hospitals and changes in the social integration of psychiatric patients. Therefore, in this study, we examined (1) whether readmissions or LoS in psychiatric wards were associated in different ways with changes in the social integration of patients with psychiatric disorders, and (2) which dimensions of social integration were the most affected.

## Methods

### Study design

This study was part of the project “Comparing policy, framework, structure, and effectiveness of Functional and Integrated systems of mental health care” (COFI), funded by the European Commission (FP7) [49]. COFI was a prospective cohort study conducted in 57 psychiatric hospitals in the UK, Poland, Germany, Italy, and Belgium. The inclusion criteria for patients were (i) being 18 years old or older, (ii) having a main diagnosis of psychotic (F20–29), mood (F30–39), or anxiety and somatoform disorder (F40–49), (iii) being hospitalised in a general adult psychiatric hospital unit, and (iv) having the ability and capacity to give informed consent. These diagnosis groups were selected as inclusion criteria to allow comparison between sites. Indeed, in the preparation of the study, we screened the diagnoses of the patients admitted to the inpatient wards and noted that these were the main diagnosis groups in all five countries. The point of entry of patients was hospitalisation from October 2014 to December 2015 in 1 of the 57 participating hospitals. Patients were followed-up 1 year after their index admission. Twenty types of inpatient and outpatient services were defined using the Client Socio-Demographic and Service Receipt Inventory–European Version CSSRI-EU [50] and data were collected on their use during the follow-up period. Although the exact availability of the existing health and social services was not assessed for each of the 57 sites, the 20 service types that were assessed were systematically provided in the different sites [51]. As a natural experiment, the COFI study addressed psychiatric patients within their natural care pathway, i.e. as it is organised in the five countries. Obviously, there were some differences between countries in terms of the whole population admitted to hospital. However, the profile of the patients included in the study was similar in all countries.

The sample size was estimated to be 1200 patients per country with an overall sample size of 6000 patients, to detect a 5% difference in readmission rates, which was the primary outcome of the study, taking into account the possible clustering effect of recruitment sites and a 15% drop-out rate [49]. The final whole sample of the study included 7302 patients with baseline and follow-up measurements available, and a pre-planned subsample of 2181 patients with more detailed follow-up measurements. Baseline data were collected via routinely collected clinical records or through face-to-face interviews with trained researchers. Patients in the pre-planned subsample were selected randomly from the whole sample and stratified according to the patients’ diagnoses and type of index admission (first admission or previous admission). The stratified random sampling was used to decrease the variance of the sample estimates [52]. The pre-planned subsample was expected to include at least 360 patients per country and 1800 patients overall [49]. The final subsample included 2181 patients, with a distribution of baseline characteristics similar to that of patients in the whole sample. Detailed follow-up data on patients’ social integration were collected from patients in the subsample via telephone contact or through face-to-face interviews 1 year after admission. This study used data from this pre-planned subsample. Ethical approvals were obtained in each of the five countries that participated in the COFI project (ref: 14/NE/1017). The detailed protocol of the COFI project has already been published elsewhere [49].

### Measures

The outcome variable was the change, over 1 year, in the patients’ level of social integration. Social integration was measured at baseline and 1 year after admission using the Objective Social Outcome Index (SIX) [53]. The SIX is a global index of social outcomes that combines different indicators of an individual’s social situation to provide a brief, meaningful, and comprehensive overview in a single indicator. The SIX index has been tested and met the following quality criteria: having a sufficient distribution to identify differences between groups, capturing changes over time to assess the potential effect of interventions, and carrying a low risk of error in assessment and documentation so that scores remain stable in the absence of real change [53]. Furthermore, The SIX was used to test the concurrent and convergent validity of other measures and scales of social integration and met the validity criteria [54, 55]. An important feature of the SIX index for this study is that it captures relevant changes in the social situation of individuals over time [53]. The SIX index ranges from 0 (low social integration) to 6 (high social integration). It includes four dimensions: employment status (0 = none, 1 = voluntary⁄protected⁄sheltered work, hereafter “protected job”, and 2 = regular employment), housing status (0 = homeless/24 h-supervised accommodation, 1 = sheltered/supported accommodation, 2 = independent accommodation), family situation (0 = living alone, 1 = living with a partner/family), and friendship status (0 = did not meet a friend in the last week, 1 = met at least one friend in the last week). A change in the overall SIX score was calculated, based on the difference between the SIX score at baseline and at follow-up to measure the change in social integration 1 year after the index admission to the hospital. Thus, the change in the SIX score ranges from -6 (major decrease in social integration) to 6 (major increase in social integration). In addition, each of the four dimensions of social integration (employment, housing, partnership and family situation, and friendship) was assessed separately. A binary variable was calculated for each dimension according to whether the score in this dimension decreased or not during the follow-up period.

The main exposure variables were the total LoS in, and the number of admissions to a psychiatric ward in general and psychiatric hospitals (i.e. acute psychiatric and long-term hospitalisations) during the follow-up period. Length of stay in hospital is a variable that is known to have a positive skewed distribution [56, 57]. We, therefore, classified the LoS into four categories using a regression tree analysis with the LoS as the dependent and independent variable. The regression tree analysis is an appropriate method for identifying cut-off points that represent the distribution of the variable in the sample and it contributes to detect possible non-linear trends and risk groups. The four LoS categories were: less than 22 days, from 22 to 75 days, from 76 to 162 days, and more than 162 days.

Several baseline socio-demographic and clinical variables which were likely to influence both LoS and social integration were included in the analysis, in line with the existing literature [7, 13, 25, 40, 41, 44, 58–60]. These were index of admission (first admission or not), involuntary admission (yes/no), age, gender, educational status, migrant status (born in the country of recruitment or not), psychiatric diagnoses (ICD-10 classification), having a comorbid diagnosis of substance misuse (yes/no), and severity of symptoms. Severity was measured using the Clinical Global Impression Scale (CGI). The CGI is a scale from 1 (normal) to 7 (among the most severely ill patients), rated by clinicians [61].

### Data analysis

Descriptive statistics were computed for patients’ socio-demographic and clinical characteristics and hospitalisation variables. Descriptive statistics were also computed according to patients’ main diagnoses (see online Supplementary Table 1). Additional descriptive statistics were computed on patients’ baseline and follow-up social integration scores to describe and model their evolution. The change in the overall SIX score had a normal distribution and met the normality criteria. Therefore, mixed-effects univariate and multivariate linear regression models were used to test the association between the change in patients’ social integration 1 year after the index admission and the total LoS, number of psychiatric admissions, patients’ characteristics, and other hospitalisation variables. As far as each social integration dimension was concerned, multivariate logistic regression models were used to test the association between each of the four dimensions of social integration and the total LoS, number of psychiatric admissions, patients’ characteristics, and other hospitalisation variables. Finally, the association between LoS and social integration may differ depending on patient profiles and on some of their socio-demographic and clinical characteristics. For example, a low level of education and a diagnosis of psychosis are both predictors of long LoS in psychiatric wards [32, 37, 62] and risk factors for social exclusion, as people with a lower level of education are more likely to be excluded from the labour market and psychotic patients are at higher risk of having a precarious housing situation [7, 60, 63]. Interactions were, therefore, computed to assess whether the main socio-demographic and clinical characteristics of patients (i.e. educational status and psychiatric diagnosis) had a moderating effect on the association between psychiatric hospitalisations and social integration. Both linear and logistic models were adjusted for country as a fixed factor, and with the hospital of admission as a random intercept. The statistical analyses were performed using SAS 9.3.

## Results

### Sample characteristics and patients’ social integration

Patients’ characteristics and variables on hospitalisation are shown in Table 1. Patients were 43 years old on average and 51% were male. The mean score of severity of patients' symptoms was 4.4 out of 7 (SD = 1.1). Thirty-six percent had a main diagnosis of psychotic disorder, 15% had a comorbid diagnosis of substance misuse, 45% had completed tertiary education, and 12% were migrants. The average total LoS in a psychiatric ward over the follow-up period was 55.6 days (SD = 62, median = 35) and the average number of psychiatric admissions was 1.6 (SD = 1.1). The index admission was the first admission for 36% of patients and 22% were admitted involuntarily at least once during the follow-up period.

**Table 1:** Study sample and characteristics

	Total sample *n* = 2181
Age, mean (SD)	43 (12)
Gender, male, *n* (%)	1114 (51)
Baseline SIX, mean (SD)	3.9 (1.4)
Follow-up SIX, mean (SD)	3.7 (1.3)
Decrease in employment status over a year, *n* (%)	283 (14)
Decrease in housing status over a year, *n* (%)	114 (6)
Decrease in family situation over a year, *n* (%)	182 (9)
Decrease in friendship status over a year, *n* (%)	335 (17)
Total length of stay in the year, mean (SD) median	55.6 (62) 35
< 22 days, *n* (%)	719 (33)
22–75 days, *n* (%)	960 (44)
76–162 days, *n* (%)	371 (17)
> 162, *n* (%)	131 (6)
Admissions in the year, mean (SD)	1.6 (1.1)
First admission, *n* (%)	783 (36)
At least one involuntary admission in the year, *n* (%)	445 (22)
Severity of symptoms (CGI), mean (SD) (1 = low, 7 = high)	4.4 (1.1)
Having a comorbid diagnosis of substance misuse, *n* (%)	289 (14.6)
Diagnosis ICD-10, *n* (%)	
Psychotic disorders	794 (36)
Mood disorders	993 (46)
Neurotic disorders	376 (17)
Others	18 (1)
Educational status, *n* (%)	
Primary	313 (15)
Secondary	858 (40)
Higher	978 (45)
Migrant status, *n* (%)	259 (12)
Country, *n* (%)	
UK	734 (34)
Italy	370 (17)
Poland	424 (19)
Germany	382 (18)
Belgium	271 (12)

Descriptive statistics were performed after exclusion of missing data

**Fig. 1:**
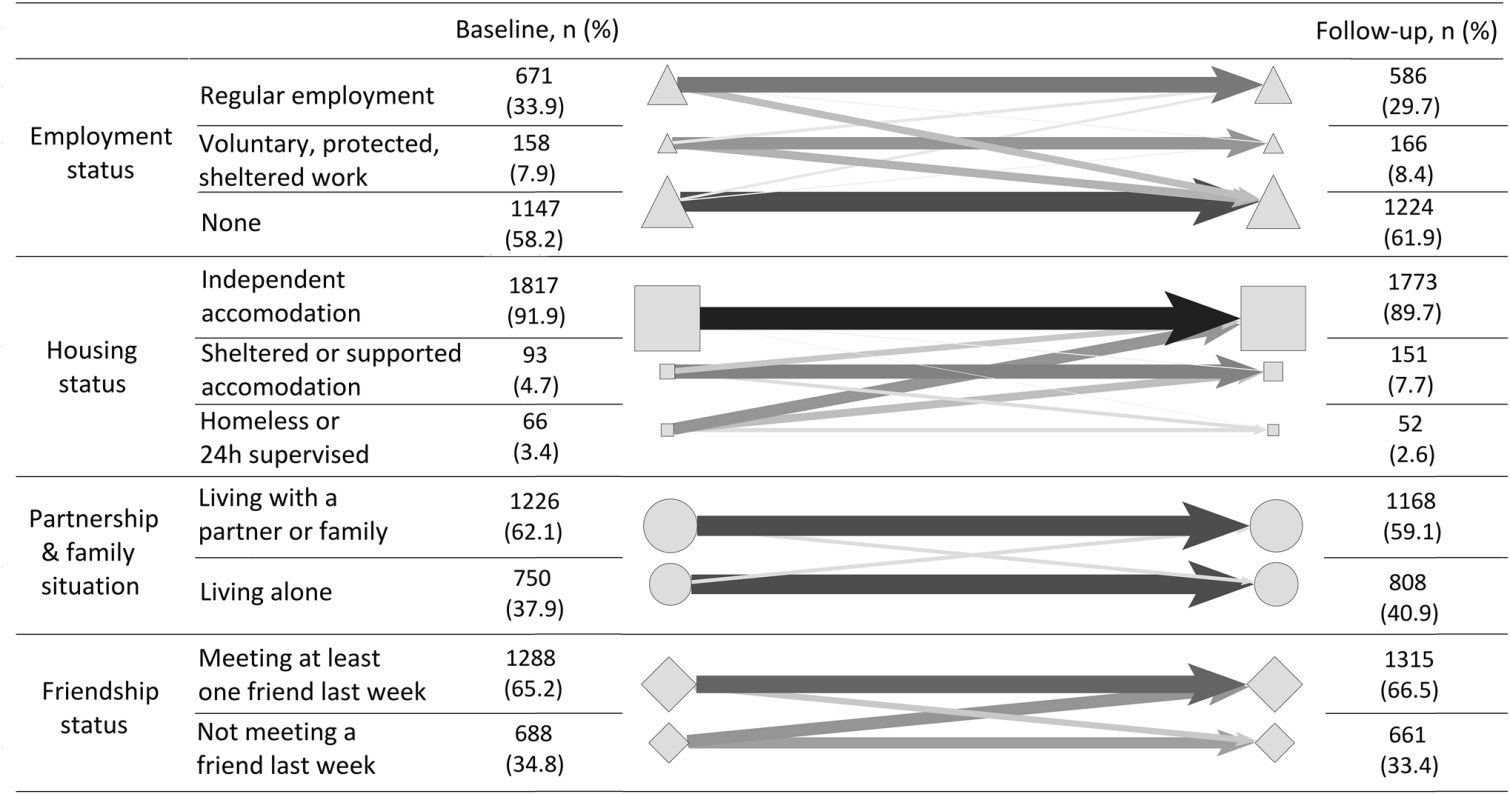
Baseline and follow-up social integration scores and change over 1 year. Shapes represent the groups of patients with the different scores in each of the four dimensions of the SIX index at baseline in the left column and at follow-up in the right column. The size of the shape is proportional to the size of the group. Arrows represent the change of score. An arrow’s width is proportional to the percentage of the group from the baseline sample. Arrows are also represented in greyscale according to the same proportions

At baseline, the average score for patients’ social integration was 3.9/6 (SD = 1.4) and it decreased slightly but significantly 1 year later: 3.7/6 (SD = 1.3) (paired t test, *t* = 4.03, *p* < 0.001). After 1 year, 14% of patients had a less favourable employment status (i.e. had become unemployed or moved from a regular to a protected job) and 6% had a less favourable housing status (i.e. had become homeless or moved from independent accommodation to sheltered or supported accommodation). Among patients who reported at baseline that they lived with a partner or that they had had contact with a friend at least once in the last week, 9% no longer live with their partner and 17% reported no such contact with a friend at follow-up. The changes in social integration scores between baseline and follow-up are presented in detail in Fig. 1. At baseline, 58.2% of patients were unemployed, 33.9% had a regular job, and 7.9% had a protected job. Between baseline and follow-up, the employment status of patients remained relatively stable, especially for unemployed patients. In terms of housing, 91.9% of patients had independent accommodation at baseline and the majority still did after 1 year. Moreover, the majority of patients who were homeless or in a 24-h supervised accommodation at baseline had independent accommodation at follow-up. The partnership and family situations of patients remained relatively stable. About 60% of patients reported living with a partner or family at baseline and at follow-up. In terms of friendship, about 65% of patients reported contact with a friend in the last week at baseline and at follow-up. The social integration scores at baseline and follow-up are shown in detail in Table 2 of the online supplementary material.

**Table 2:** Association between hospitalisation in psychiatric inpatient units and the change in social integration, employment, housing, friendship status, and family situation of psychiatric patients over a year

	Change in social integration over a year		Decrease in employment status over a year		Decrease in housing status over a year		Decrease in family situation over a year		Decrease in friendship status over a year	
	*β*	*P* value	OR	*P* value	OR	*P* value	OR	*P* value	OR	*P* value
Length of stay										
< 22 days	REF	REF	REF	REF	REF	REF	REF	REF	REF	REF
22–75 days	− 0.01	0.95	1.09	0.68	0.91	0.34	1.37	0.12	1.10	0.95
76–162 days	− 0.12	0.07	1.41	0.06	2.34	0.005	1.57	0.08	1.008	0.61
> 162 days	− 0.23	0.03	2.21	0.02	3.45	< 0.001	1.94	0.04	1.10	0.63
Number of admissions	− 0.05	0.54	1.006	0.31	0.90	0.33	0.98	0.99	1.15	0.03
First admission (Yes)	− 0.08	0.83	1.42	0.01	0.52	0.01	1.08	0.11	0.97	0.79
Involuntary admission (Yes)	0.03	0.50	0.99	0.97	1.02	0.92	0.75	0.33	0.96	0.62

Multivariate linear and logistic regression models adjusted for variables in the models and age, gender, severity of symptoms, psychiatric diagnosis, comorbid diagnosis of substance misuse, educational status, migrant status, and country as fixed factors and the admission hospital as a random intercept

### Association between hospitalisations in psychiatric wards and the change in patients’ level of social integration after 1 year

The associations between the change in patients’ social integration over 1 year and variables of hospitalisation are shown in Table 2. In multivariate analysis, a longer total LoS in psychiatric wards was significantly associated with a decrease in the social integration score, but not the other hospitalisation variables. The negative association between LoS and patients’ social integration scores was dose–response and significant for patients who were hospitalised for more than 162 days during the follow-up period (*β* = − 0.23, *p* = 0.03).

In particular, employment, family, and housing status significantly decreased with a longer LoS. Compared to patients who were hospitalised for less than 22 days over the follow-up period, patients who were hospitalised for more than 162 days were more likely to become unemployed or to move from a regular to a protected job (OR = 2.21, *p* = 0.02) and more likely to no longer live with their partner or family (OR = 1.94, *p* = 0.04). Patients who stayed in hospital for between 76 and 162 days (OR = 2.34, *p* = 0.005) and for more than 162 days (OR = 3.45, *p* < 0.001) were also more likely to become homeless or to move from independent accommodation to sheltered or supported accommodation during the follow-up period than patients whose total LoS was lower than 22 days.

There was no significant association between LoS and friendship status. However, friendship status significantly decreased with a higher number of admissions over the follow-up period (OR = 1.15, *p* = 0.03). In contrast, there was no association between the number of admissions and the other dimensions of social integration.

Finally, patients who had been hospitalised for the first time in a psychiatric ward were more likely to become unemployed or to move from a regular to a protected job (OR = 1.42, *p* = 0.01). They were, however, less likely to become homeless or to move from independent accommodation to sheltered or supported accommodation (OR = 0.52, *p* = 0.01) than patients who had been admitted previously.

### The temporal relation between length of stay in psychiatric wards and the change in patients’ level of social integration after 1 year

As the LoS in psychiatric wards and the patients’ level of social integration are likely to affect each other, a sensitivity analysis was performed using the same statistical model as in Table 2, but including only the LoS of the index admission, instead of the total LoS during the follow-up period. The objective of this analysis was to gain more insight into the temporal relation between LoS and the change in social integration, as the LoS of the index admission occurred before the change in social integration over the follow-up period. The results showed a significant, negative association between the LoS of the index admission and the change in social integration over the follow-up period (*β* = − 0.14, *p* = 0.03).

### Socio-demographic and clinical moderators of the association between length of stay, number of admissions in a psychiatric ward, and the change in the patient’s level of social integration after 1 year

The socio-demographic and clinical moderators are shown in Tables 3, 4. The patient’s main psychiatric diagnosis and educational status were significant moderators of the association between LoS and the level of social integration in the multivariate analysis. Patients with a main diagnosis of mood disorder who were hospitalised for more than 76 days during the study period were significantly more likely to have a decrease in their social integration score (76–162 days, *β* = − 0.52, *p* = 0.01; > 162 days, *β* − 0.81, *p* = 0.004) than patients with a main diagnosis of psychotic disorder who had the same LoS. In particular, a decrease was found in their employment status (76–162 days, OR = 2.08, *p* = 0.04; > 162 days, OR = 4.13, *p* = 0.01). Patients with a mood disorder and a long LoS, however, were less likely to have their housing status decrease (76–162 days, OR = 0.45, *p* = 0.01; > 162 days, OR = 0.38, *p* = 0.008) than patients with a main diagnosis of psychotic disorder who had the same LoS. Patients who had a low level of education (i.e. primary education) who were hospitalised for between 76 and 162 days during the study period were less likely to experience a decrease in their employment status (OR = 0.65, *p* = 0.02) than patients with a higher level of education (i.e. tertiary education) with the same LoS. However, patients with a lower level of education who were hospitalised for more than 76 days were more likely to experience a decrease in their housing status (76–162 days, OR = 2.63, *p* = 0.03; > 162 days, OR = 4.76, *p* = 0.007) than patients with a higher level of education and the same LoS. The other socio-demographic and clinical characteristics were not significant moderators.

**Table 3:** Socio-demographic and clinical moderators of the association between length of stay, number of admissions in psychiatric wards, and changes in patients’ social integration outcomes over the follow-up period

	Change in social integration over a year				Decrease in employment status over a year			
	Main effect *Β* (*p* value)	Interaction *β* (*p* value)			Main effect OR (*p* value)	Interaction OR (*p* value)		
		Length of stay (days) ^c^				Length of stay (days) ^**c**^		
		22–75	76–162	> 162		22–75	76–162	> 162
Diagnosis ICD-10^a^								
Mood disorders	− 0.17 (0.25)	− 0.21 (0.65)	− 0.52 (0.01)	− 0.81 (0.004)	1.75 (0.02)	1.32 (0.19)	2.08 (0.04)	4.13 (0.01)
Neurotic disorders	− 0.05 (0.81)	− 0.03 (0.54)	− 0.17 (0.63)	− 0.23 (0.29)	1.09 (0.52)	1.12 (0.53)	1.28 (0.16)	1.08 (0.79)
Educational status^b^								
Primary	0.11 (0.59)	− 0.08 (0.93)	0.09 (0.71)	0.007 (0.95)	0.78 (0.006)	0.92 (0.65)	0.65 (0.02)	0.78 (0.13)
Secondary	0.09 (0.72)	0.04 (0.81)	− 0.02 (0.79)	0.19 (0.31)	0.98 (0.93)	0.97 (0.83)	0.72 (0.53)	0.89 (0.68)
Length of stay (days)^c^								
> 162	− 0.20 (0.04)	/	/	/	2.11 (0.03)	/	/	/
76–162	− 0.09 (0.52)	/	/	/	1.11 (0.08)	/	/	/
22–75	− 0.02 (0.76)	/	/	/	1.01 (0.88)	/	/	/
Number of admissions	− 0.02 (0.15)	/	/	/	0.89 (0.78)	/	/	/

Multivariate linear and logistic regression models adjusted for variables in the model and age, gender, severity of symptoms, comorbid diagnosis of substance misuse, migrant status, first admission, involuntary admission, country as fixed factors and the admission hospital as a random intercept

^a^Reference category = psychotic disorders, ^b^Reference category = tertiary or further education, ^c^Reference category =  < 22 days

**Table 4:** Socio-demographic and clinical moderators of the association between length of stay, number of admissions in psychiatric wards, and changes of patients’ social integration outcomes over the follow-up period

	Decrease in housing status over a year				Decrease in family situation over a year				Decrease in friendship status over a year	
	Main effect OR (*p* value)	Interaction OR (*p* value)			Main effect OR (*p* value)	Interaction OR (*p* value)			Main effect OR (*p* value)	Interaction OR (*p* value)
		Length of stay (days) ^c^				Length of stay (days) ^c^				No. of admissions
		22–75	76–162	> 162		22–75	76–162	> 162		
Diagnosis ICD-10^a^										
Mood disorders	0.41 (0.009)	0.71 (0.32)	0.45 (0.01)	0.38 (0.008)	1.02 (0.92)	0.47 (0.21)	0.51 (0.29)	0.89 (0.73)	0.89 (0.81)	1.02 (0.85)
Neurotic disorders	0.82 (0.63)	0.71 (0.32)	1.13 (0.53)	0.59 (0.29)	2.04 (0.07)	0.89 (0.73)	1.12 (0.54)	1.81 (0.09)	0.78 (0.67)	1.05 (0.76)
Educational status^b^										
Primary	1.42 (0.01)	0.97 (0.81)	2.63 (0.03)	4.76 (0.007)	1.71 (0.07)	1.47 (0.68)	1.68 (0.61)	1.76 (0.11)	1.43 (0.12)	1.35 (0.29)
Secondary	1.02 (0.89)	1.05 (0.96)	1.22 (0.18)	1.38 (0.11)	0.96 (0.83)	0.91 (0.85)	0.98 (0.87)	0.61 (0.31)	0.98 (0.86)	0.89 (0.76)
Length of stay (days)^c^										
> 162 days	1.82 (0.01)	/	/	/	1.76 (0.04)	/	/	/	/	/
76–162 days	1.59 (0.04)	/	/	/	1.31 (0.24)	/	/	/	/	/
22–75 days	1.03 (0.87)	/	/	/	1.14 (0.43)	/	/	/	/	/
Number of admissions	0.93 (0.89)	/	/	/	0.94 (0.85)	/	/	/	1.11 0.04	/

Multivariate logistic regression models adjusted for variables in the model and age, gender, severity of symptoms, comorbid diagnosis of substance misuse, migrant status, first admission, involuntary admission, country as fixed factors and the admission hospital as a random intercept

^a^Reference category = psychotic disorders, ^b^Reference category = tertiary or further education, ^c^Reference category =  < 22 days

## Discussion

### Main findings

Having a long length of stay in a psychiatric ward was more strongly associated with a decrease in the social integration of psychiatric patients after 1 year than experiencing repeated admissions. The dimensions of social integration that were more strongly and negatively associated with longer lengths of stay were the patients’ housing, employment, and household living situation. Psychiatric patients who experienced a total of more than 76 days of psychiatric hospitalisation in 1 year had a significantly higher probability of becoming homeless or moving from independent accommodation to sheltered or supported accommodation. Moreover, psychiatric patients who were hospitalised for more than 162 days in 1 year had a significantly higher probability of becoming unemployed or moving from a regular job to a form of protected job. There was also a higher probability that patients who were hospitalised for more than 162 days would no longer be living with their partner or family. In contrast, repeated admissions over 1 year was not associated with such a decrease in employment, accommodation, and family status, though it was negatively associated with friendship contact. In terms of housing, patients who were hospitalised for the first time were less likely to be affected than patients who had previously been admitted to hospital.

The main psychiatric diagnosis and the educational status of the patient were moderators of the association between LoS and the change in social integration, particularly in relation to employment and housing status. Among patients who have been staying in hospital for more than 76 days over 1 year, those with a main diagnosis of mood disorder and those with a higher level of education were more likely to experience a decrease in their employment status. In contrast, patients with a main diagnosis of psychotic disorder were more likely to experience a decrease in their housing status. Among patients who have been staying in hospital for more than 162 days, those with a lower level of education were more likely to experience a decrease in their housing status.

### Interpretation of findings

So far, the literature on the association between hospitalisation in psychiatric wards and patients’ social integration has been inconclusive [25, 47, 48]. The results of the present study are, however, consistent with the literature suggesting that a longer length of stay in a psychiatric ward is associated with a decrease in the social integration of psychiatric patients, in particular on dimensions such as employment and housing [44]. Obviously, the relationship between the LoS in hospital or hospital readmissions and patients’ level of social integration is complex, as they are likely to affect each other. Given the design of the study, we cannot disentangle the temporal relation found between LoS and the change in the social integration of patients over the study period. A long LoS in a psychiatric ward might disrupt a patient’s social and professional life and negatively affect his/her social integration, but a decrease in a patient’s level of social integration might also cause a relapse and, consequently, a longer LoS in hospital. The latter interpretation, however, should not be overstated for two main reasons. First, some studies have shown that patients’ socio-demographic and clinical characteristics only weakly predict the LoS in psychiatric hospitals [32, 38]. A recent study found that patients’ characteristics, including clinical status, predicted only 15% of the variance of LoS in psychiatric hospitals [37]. Authors have argued that, although poor social functioning may reflect the need for lengthy hospitalisation, the possibility that lengthy stays lead to poor social functioning should also be taken into account and have appealed for more research into these aspects. Second, the results of the sensitivity analysis of the association between the LoS of the index admission and the change in social integration showed that having a long length of stay during the index admission was significantly associated with a decrease in the social integration of psychiatric patients after 1 year. Taken together, these two arguments support the hypothesis that long LoS in psychiatric wards have an effect on patients’ social integration.

The study also found that a long length of stay is more strongly associated with a decrease in the social integration of patients than repeated admissions. This finding has important practical implications for policies and interventions. To the best of our knowledge, this result has never been highlighted before. This finding suggests that a reduction of the length of hospital stays is consistent with policies and interventions aiming to strengthen patients’ social integration. Some authors have argued that the process of deinstitutionalisation and the reduction of LoS in psychiatric hospitals have had adverse effects on patients’ social integration with, for example, an increase in homelessness and social isolation [44, 64–66]. The results of the present study, however, indicate that the relationship between length of hospital stay and social integration is more complex and that, to a certain extent, long stays in psychiatric wards may also have adverse effects on social integration.

Patients’ friendship was the only dimension of social integration that was negatively associated with repeated hospital admissions. Although further research would be needed to explore and understand this association more in detail, one possible interpretation is that creating and maintaining friendship ties takes time and is probably more difficult when a patient moves back and forth between hospital and the community. In addition, this finding is consistent with other studies examining the social support network of psychiatric patients [67].

This study also highlights some clinical and socio-demographic risk factors related to social exclusion and, more specifically, to job and housing loss during long stays in psychiatric wards, that care providers should consider more carefully. Patients with a main diagnosis of mood disorder who were hospitalised for a long period were more likely to experience a decrease in their overall social integration. People with mood disorders are known to have relatively better social integration than people with psychotic disorders [18, 19]. One possible interpretation of this result is that people with a diagnosis of mood disorder and a higher level of social integration at baseline are more likely to experience a decrease in social integration during a long hospitalisation than people with a lower level of social integration at baseline. Regarding job loss, being admitted for the first time, having a main diagnosis of mood disorder, and having a high level of education were risk factors. People with a high education status are more likely to work within a competitive environment. Experiencing their first, long psychiatric hospitalisation with a diagnosis of psychiatric disorder, therefore, has heavier consequences for their employment. In contrast, having had several previous hospitalisations, having a main diagnosis of psychotic disorder, and having a low level of education were risk factors in terms of housing loss. Patients with a lower educational status may have a more unstable housing situation and are, therefore, more likely to be affected by long hospital stays in terms of accommodation. Psychotic patients are at a high risk of homelessness or of having a precarious housing situation [13, 60]. However, if a longer stay in hospital may seem appropriate for psychotic patients in precarious housing situations and without accommodation, it can be detrimental for those who have an independent accommodation solution.

### Strengths and limitations

In a review conducted in 2014 that compared the effects of short and long hospital stays on the social functioning and social integration of psychiatric patients, the most recent study included was from 1980 and the largest sample size was 1169 patients [44]. The main strength of this study is, therefore, its large sample of more than 2000 patients recruited from 57 hospitals in 5 European countries. Because it is so large and diverse, that sample provides not only a high statistical power but also a strong external validity to different contexts and countries.

This study, however, also has some limitations. One major limitation is that the association between LoS and readmissions in a psychiatric ward and patients’ social integration is likely to be affected by several other confounding and unobserved variables, the most important of which is probably the clinical status of the patient. Given the design of the study, i.e. a natural experiment, the inclusion of patients across the 57 hospitals was not random and there were potential confounding factors. A causal relationship between LoS or readmissions in psychiatric ward and a change in social integration cannot, therefore, be determined. Only a randomised clinical trial (RCT), in which the LoS and the frequency of hospitalisation were randomised would make it possible to establish causation. However, randomising the LoS and the frequency of hospitalisation entails other obstacles and may be clinically and ethically problematic. The few RCTs published in the second half of the twentieth century did not randomise the LoS, but randomly allocated patients to different types of hospitalisation, i.e. planned short-stay admission or brief hospitalisation versus long or standard stay [48, 68–72]. However, it is difficult to draw conclusions about the impact of LoS on the social integration of patients, because the results of these studies were inconsistent and because the definition of a short stay admission varied from one study to another. Moreover, the patients allocated to short-stay hospitalisations also received other treatments, such as discharge planning or intensive aftercare. By contrast, in our study, this risk of confounding bias was partially overcome, because analyses were controlled for several baseline variables known to be associated with both LoS and readmissions and social integration, and were adjusted with the hospital as a random intercept to take into account a potential clustering effect. We cannot rule out, however, that other possible confounding and unobserved factors were not taken into account, e.g. the physical health status of patients and the evolution of the psychiatric disorder during the follow-up period. A sensitivity analysis was, therefore, performed to test the endogeneity of LoS in relation to social integration. The null hypothesis was not rejected (LR = 0.76, *p* = 0.38), indicating the absence of endogeneity of LoS to unobserved confounders affecting social integration [73, 74]. This result suggests that the variation of LoS is not likely to be related to unobserved confounders, e.g. unobserved clinical or socio-demographic characteristics of patients.

Another limitation is related to the sample selection. We cannot rule out a selection bias, as the study is based on data from a pre-planned subsample. To control for this potential bias, we examined the difference between the baseline social integration scores of patients in the subsample compared to the other patients in the whole sample. The mean score in the subsample was 3.87/6 (SD = 1.39), while it was 3.69/6 (SD = 1.43) for the other patients in the whole sample (*t* test = 2.46, *p* = 0.01). Although the difference was statistically significant, it was very small and, therefore, very unlikely to have clinical or social significance. In addition, the risk of selection bias was minimised because the pre-planned subsample was randomly selected from the whole sample. Finally, social integration is a multidimensional concept. Although the SIX index has been validated [53], it aims to summarise a complex phenomenon using a limited number of dimensions. For example, information about social relationships and support is probably not entirely captured by a single question about the frequency of meeting a friend. Therefore, other dimensions of social integration might be worth studying in this context, e.g. social participation and political and community engagement.

## Conclusion

The social integration of patients with psychiatric disorders is a major objective of mental health systems, policies, and services, both for clinicians and for patients themselves [2, 4, 11]. This study supports the importance of policies and interventions that aim to reduce the length of hospital stays for psychiatric patients to preserve their social integration. The results of this study suggest that shorter lengths of stay in hospitals should be favoured, especially for patients with mood disorders, not so much from a budgetary perspective, but to protect patients’ employment, housing, and partnership-family situations, and that fewer hospital stays should be favoured to protect patients’ friendships. In addition, special attention should be paid to helping psychiatric patients to find and retain their housing and employment while hospitalised. Therefore, the results of this study also support the importance of evidence-based employment and housing support interventions for psychiatric patients, e.g. Housing First and Individual Placement and Support [75–77].

The objective of this study was not to make cross-country comparisons. In view of the differences between countries in terms of length of stay, mental health care, and social integration policies, however, further research might compare the association between long LoS or readmissions in psychiatric wards and the social integration of psychiatric patients in different countries [32, 78, 79]. Finally, a one-year follow-up period is relatively short to detect significant and meaningful changes in the social integration of individuals. Further studies might use a comparable design with a longer follow-up period, especially since we know that psychiatric patients sometimes use psychiatric hospitals throughout their lifetime.

## Electronic supplementary material

Below is the link to the electronic supplementary material.

Supplementary file1 (DOCX 16 kb)

Supplementary file2 (DOCX 16 kb)
