# Supplementary material for: Use of psychiatric hospitals and social integration of patients with psychiatric disorders: a prospective cohort study in five European countries
Source: Soc Psychiatry Psychiatr Epidemiol. 2020 May 14;55(11):1425–38. doi: 10.1007/s00127-020-01881-1 (PMC7578147; doi:10.1007/s00127-020-01881-1)
Supplement: Supplementary file 1 — Supplementary file1 (DOCX 16 kb) [file 127_2020_1881_MOESM1_ESM.docx]

Online Supplementary Table 1: Study sample and characteristics according to the main diagnosis groups

|  | **Psychotic disorders**  **n = 794** | **Mood**  **disorders**  **n = 993** | **Neurotic**  **disorders**  **n = 376** | **Khi^2^**  **ANOVA**  **(p-value)** |
| --- | --- | --- | --- | --- |
| Age, mean (SD) | 39.4 (12.1) | 43.2 (12.1) | 39.2 (12.5) | 25.4 (<0.001) |
| Gender, male, n (%) | 438 (62.7) | 414 (44.6) | 161 (46.3) | 56.5 (<0.001) |
| Baseline social integration score (SIX), mean (SD) | 3.5 (1.3) | 4.3 (1.3) | 4.3 (1.4) | 58.9 (<0.001) |
| Follow-up social integration score (SIX), mean (SD) | 3.3 (1.3) | 4.1 (1.3) | 4.2 (1.4) | 58.2 (<0.001) |
| Decrease in employment status over a year, n (%) | 87 (10.9) | 151 (15.2) | 45 (11.8) | 10.1  (0.006) |
| Decrease in housing status over a year, n (%) | 70 (10.0) | 30 (3.2) | 14 (4.0) | 36.1 (<0.001) |
| Decrease in family situation over a year, n (%) | 64 (9.1) | 77 (8.3) | 41 (11.8) | 3.6  (0.15) |
| Decrease in friendship status over a year, n (%) | 121 (17.3) | 152 (16.4) | 62 (17.8) | 0.5  (0.78) |
| Total length of stay in the year, mean (SD) median   - < 22 days, n (%) - 22 – 75 days, n (%) - 76 – 162 days, n (%) - > 162, n (%) | 63.9 (71) *41*  195 (27.9)  316 (45.3)  133 (19.1)  53 (7.6) | 54.4 (56.1) *36*  173 (31.1)  430 (46.3)  161 (17.3)  48 (5.2) | 41.9 (55) *23*  173 (49.7)  123 (35.3)  34 (9.7)  18 (5.1) | 15.1 (<0.001)  59.8  (<0.001) |
| Admissions in the year, mean (SD) | 1.7 (1.0) | 1.7 (1.2) | 1.6 (1.1) | 0.3  (0.76) |
| First admission, n (%) | 162 (23.2) | 350 (37.7) | 202 (58.1) | 124.1 (<0.001) |
| At least one involuntary admission in the year, n (%) | 221 (31.6) | 180 (19.4) | 38 (10.9) | 65.7 (<0.001) |
| Severity of symptoms (CGI), mean (SD)  (1 = low, 7 = high) | 4.5 (1.1) | 4.3 (1.1) | 3.9 (1.1) | 51.3 (<0.001) |
| Having a comorbid diagnosis of substance misuse, n (%) | 117 (16.7) | 119 (12.8) | 53 (15.2) | 5.1  (0.07) |
| Educational status, n (%)   - Primary - Secondary - Higher | 102 (14.6)  304 (43.5)  293 (41.9) | 117 (12.6)  347 (37.4)  464 (50.0) | 61 (17.5)  140 (40.2)  147 (42.2) | 15.1  (0.004) |
| Migrant status, n (%) | 101 (14.4) | 111 (11.9) | 27 (7.8) | 9.8  (0.007) |

Descriptive statistics were performed after exclusion of missing data.
