# Supplementary material for: Use of psychiatric hospitals and social integration of patients with psychiatric disorders: a prospective cohort study in five European countries
Source: Soc Psychiatry Psychiatr Epidemiol. 2020 May 14;55(11):1425–38. doi: 10.1007/s00127-020-01881-1 (PMC7578147; doi:10.1007/s00127-020-01881-1)
Supplement: Supplementary file 2 — Supplementary file2 (DOCX 16 kb) [file 127_2020_1881_MOESM2_ESM.docx]

Online Supplementary Table 2: Baseline and follow-up measures of social integration

|  | | Baseline scores | Follow-up  Scores | | | Total  n (%) |
| --- | --- | --- | --- | --- | --- | --- |
|  |  |  | 2 | 1 | 0 |  |
| Employment status  n (%) | Regular employment | 2 | 443 (66.1) | 25 (3.7) | 203 (30.2) | 671 (100) |
|  | Voluntary, protected, sheltered work | 1 | 20 (12.7) | 83 (52.5) | 55 (34.8) | 158 (100) |
|  | None | 0 | 123 (10.7) | 58 (5.1) | 966 (84.2) | 1147 (100) |
| Housing status  n (%) | Independent accommodation | 2 | 1717 (95.5) | 73 (4.0) | 27 (1.5) | 1817 (100) |
|  | Sheltered or supported accommodation | 1 | 23 (24.7) | 56 (60.2) | 14 (15.1) | 93 (100) |
|  | homeless or 24 h supervised | 0 | 33 (50.0) | 22 (33.3) | 11 (16.7) | 66 (100) |
| Partnership  & family situation  n (%) | Living with a partner or family | 1 | / | 1044 (85.2) | 182 (15.8) | 1226 (100) |
|  | Living alone | 0 | / | 124 (16.5) | 626 (83.5) | 750 (100) |
| Friendship status  n (%) | Meeting at least one friend last week | 1 | / | 953 (74.0) | 335 (26.0) | 1288 (100) |
|  | Not meeting a friend last week | 0 | / | 362 (52.6) | 326 (47.4) | 688 (100) |
